# Availability of medical abortion medicines in eight countries: a descriptive analysis of key findings and opportunities

**DOI:** 10.1186/s12978-023-01574-3

**Published:** 2023-04-11

**Authors:** Amy Grossman, Ndola Prata, Natalie Williams, Bela Ganatra, Antonella Lavelanet, Laurence Läser, Chilanga Asmani, Hayfa Elamin, Leopold Ouedraogo, Md. Mahmudur Rahman, Musu Julie Conneh-Duworko, Bentoe Zoogley Tehoungue, Harriet Chanza, Henry Phiri, Bharat Bhattarai, Narayan Prasad Dhakal, Olumuyiwa Adesanya Ojo, Kayode Afolabi, Theopista John Kabuteni, Binyam Getachew Hailu, Francis Moses, Sithembile Dlamini-Nqeketo, Thembi Zulu, Ulrika Rehnström Loi

**Affiliations:** 1 Venture Strategies for Health & Development/OASIS, Berkeley, CA USA; 2 Bixby Center for Population, Health & Sustainability, School of Public Health, University of California, Berkeley, CA USA; 3 UNDP-UNFPA-UNICEF-WHO-World Bank Special Programme of Research, Development and Research Training in Human Reproduction (HRP), Department of Sexual and Reproductive Health and Research, World Health Organization, 20 Avenue Appia, 1211 Geneva, Switzerland; 4 World Health Organization, Regional Office for Africa, Brazzaville, Republic of Congo; 5 Directorate General of Family Planning, Karwarbazar, Dhaka, Bangladesh; 6 World Health Organization, Liberia Country Office, Monrovia, Republic of Liberia; 7 Family Health Program, Ministry of Health, Monrovia, Republic of Liberia; 8 World Health Organization, Malawi Country Office, Lilongwe, Republic of Malawi; 9 Ministry of Health, Lilongwe, Republic of Malawi; 10 Department of Drug Administration, Ministry of Health and Population, Kathmandu, Nepal; 11 Ministry of Health and Population, Kathmandu, Nepal; 12 World Health Organization, Nigeria Country Office, Abuja, Federal Republic of Nigeria; 13 Reproductive Health, Federal Ministry of Health, Abuja, Federal Republic of Nigeria; 14 World Health Organization, Rwanda Country Office, Kigali, Republic of Rwanda; 15 World Health Organization, Sierra Leone Country Office, Freetown, Sierra Leone; 16 Reproductive Health/Family Planning Programme Manager, Ministry of Health, Freetown, Sierra Leone; 17 World Health Organization, South Africa Country Office, Pretoria, Republic of South Africa; 18 National Department of Health, Pretoria, Republic of South Africa

**Keywords:** Medical abortion, Mifepristone, Misoprostol, Combi-pack, Abortion

## Abstract

**Background:**

In recent years a growing number of manufacturers and medical abortion products have entered country markets and health systems, with varying degrees of quality and accessibility. An interplay of factors including pharmaceutical regulations, abortion laws, government policies and service delivery guidelines and provider’s knowledge and practices influence the availability of medical abortion medicines. We assessed the availability of medical abortion in eight countries to increase understanding among policymakers of the need to improve availability and affordability of quality-assured medical abortion products at regional and national levels.

**Methods:**

Using a national assessment protocol and an availability framework, we assessed the availability of medical abortion medicines in Bangladesh, Liberia, Malawi, Nepal, Nigeria, Rwanda, Sierra Leone and South Africa between September 2019 and January 2020.

**Results:**

Registration of abortion medicines—misoprostol or a combination of mifepristone and misoprostol—was established in all countries assessed, except Rwanda. Mifepristone and misoprostol regimen for medical abortion was identified on the national essential medicines list/standard treatment guidelines for South Africa as well as in specific abortion care service and delivery guidelines for Bangladesh, Nepal, Nigeria, and Rwanda. In Liberia, Malawi, and Sierra Leone—countries with highly restrictive abortion laws and no abortion service delivery guidelines or training curricula—no government-supported training on medical abortion for public sector providers had occurred. Instead, training on medical abortion was either limited in scope to select private sector providers and pharmacists or prohibited. Community awareness activities on medical abortion have been limited in scope across the countries assessed and where abortion is broadly legal, most women do not know that it is an option.

**Conclusion:**

Understanding the factors that influence the availability of medical abortion medicines is important to support policymakers improve availability of these medicines. The landscape assessments documented that medical abortion commodities can be uniquely impacted by the laws, policies, values, and degree of restrictions placed on service delivery programs. Results of the assessments can guide actions to improve access.

## Plain Language Summary

Unsafe abortion is a leading cause of death and disability among women of reproductive age. Medical management of abortion with mifepristone and misoprostol pills, or just misoprostol, is a safe and effective way to end a pregnancy. Owing to an increase in the number of medical abortion products that have entered country health systems, we examined access to these medicines from supply to demand in selected countries. The overarching goal of the national landscape assessments was to produce evidence to support advocacy efforts and policymaking for improved access to quality medical abortion products that is appropriate to the needs of the country. This paper aims to describe key findings across eight country settings on the availability of medical abortion medicines and identify key opportunities to improve access to them across countries.

## Background

Despite being largely preventable, unsafe abortion—an abortion carried out by a person lacking necessary skills or in an environment that lacks minimal medical standards, or both—remains a leading cause of maternal mortality and morbidity [1, 2]. Restrictive abortion laws and policies, stigma and other barriers, drive women to induce abortion themselves using unsafe methods or seek abortion from unskilled providers, contributing to unsafe abortion [1–3]. Estimates from 2010 to 2014 suggest that about 45% of all abortions were unsafe and nearly all took place in a developing country [1]. In addition to risk of severe disability or death for women in developing countries, the management of abortion complication places a burden on healthcare systems [4]. The use of medical abortion (MA) using either a combination of mifepristone followed by misoprostol, or misoprostol alone has contributed to increased safety and decreased mortality and morbidity [5]. MA can be effectively and safely administered at a healthcare facility by differing levels of healthcare providers or self-administered for abortions less than 12 weeks outside of a facility by individuals with accurate information and quality-assured medicines [2].

Access to quality-assured MA medicines, including mifepristone, misoprostol and co-packaged mifepristone and misoprostol (combi-pack), plays a critical role in providing safe abortion care. In recent years, the number of misoprostol-alone and combi-pack products that have been registered for obstetric and gynaecologic indications has grown globally [6–10]. Understanding the factors that influence the availability of MA medicines is important to help policymakers, program planners, and providers in countries improve availability and use of quality medicines.

This paper describes the World Health Organization’s landscape assessments on the availability of MA medicines including the combi-pack, mifepristone and misoprostol in Bangladesh, Liberia, Malawi, Nepal, Nigeria, Rwanda, Sierra Leone, and South Africa. The purpose was to document country-specific experiences related to MA medicines availability and use, with a focus on the combi-pack, and define opportunities for improved access.

## Methods

The country selection criteria were based on discussions with WHO Regional Office and HRP/WHO SRH Department technical staff. Factors such as opportunity to increase access for MA medicines, experience of conducting relevant work in the country and country request were considered during the selection process. Bangladesh, Liberia, Malawi, Nepal, Nigeria, Rwanda, Sierra Leone, and South Africa were selected for the initial round of national assessments. In addition to the above considerations, these eight countries were chosen due to their public health needs and because they offered varying legal frameworks for abortion under which to assess MA medicine availability.

**Fig. 1:**
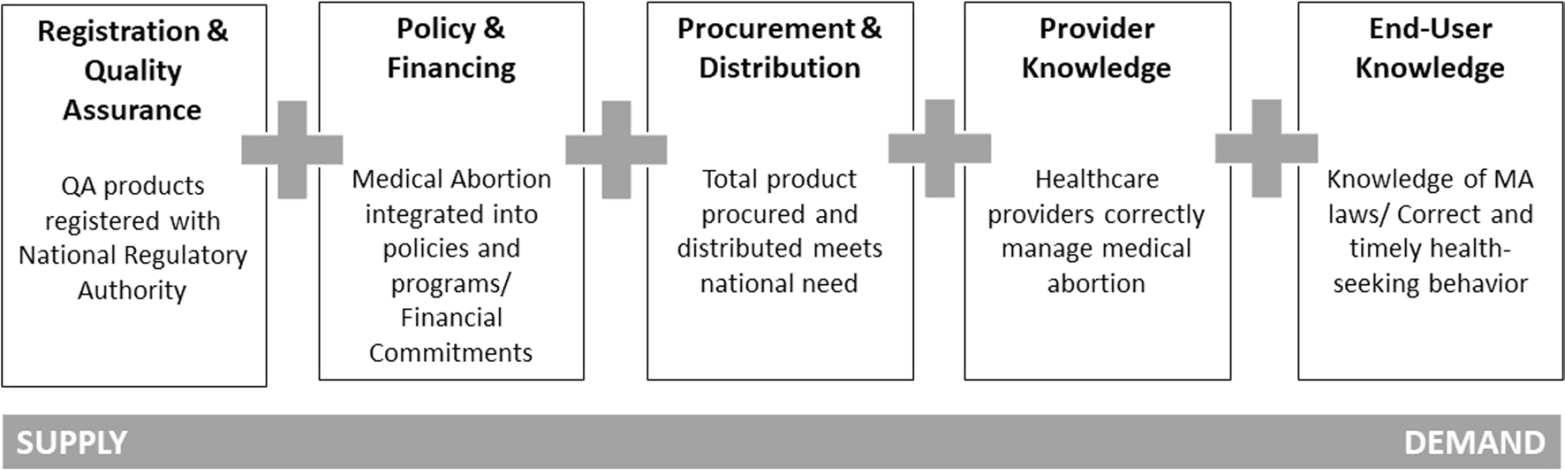
The five pillars of availability of a medical product related service, applied to MA. Source: Rehnstrom Loi et al. [11]

We developed a country assessment protocol to guide the methodology of the national landscape assessments [11]. The assessment protocol included adaptation of an availability framework, a desk review that included a literature review and online data gathering, country-level key informant interviews, and analysis of the data to identify barriers and opportunities to improve MA availability. The assessments were part of a programme and not research per se. However, consent to participate in providing information was asked to all participants. The assessments and its findings were organized around the availability framework composed of five areas or “pillars” that span all aspects of availability and use of a medicine, from supply by the manufacturer to demand and use by the end user (Fig. 1). Each pillar has a set of conditions that should be fulfilled to ensure availability, and a series of indicators to determine how well those conditions are being met. Data was collected between September 2019 and January 2020.

## Results

### Registration and quality assurance

MA products that were registered by the national regulatory authority (NRA) in each of the eight program countries are shown in Table 1. For the assessment, the category of abortion law restriction was evaluated following a reading of each country’s abortion law, from low restriction (abortion upon request) to high (abortion only to save the life of the woman). For the purpose of these assessments, a quality-assured product is defined as one that is either WHO Prequalification (WHO-PQ)-listed or approved by a Stringent Regulatory Authority (SRA). Rwanda had no MA medicines registered whereas Bangladesh had the greatest number of registered MA medicines; given its domestic manufacturing capacity, however only one product was quality-assured (Misoclear®, Acme Pharmaceuticals). A quality-assured combi-pack (Medabon®, Sun Pharmaceuticals) was registered in only one country, Nepal. In four other countries, combi-packs that are neither WHO PQ-listed or SRA-approved were registered and were most commonly made by the Indian manufacturers Acme Formulations and Naari. We found that the WHO’s Collaborative Registration Procedure, which can enable accelerated regulatory approval, was underutilized in all countries assessed. We found that when reliance mechanisms such as this and other fast-track mechanisms are used, regulatory approval of MA products can take 90 days, but up to 5 years otherwise.

**Table 1:** Registration and quality assurance status of MA medicines by country and abortion law restrictions

Country	Abortion Law level of restriction	Combi-pack		Misoprostol		Mifepristone	
		Number of registered products	Number quality-assured products	Number of registered products	Number quality-assured products	Number of registered products	Number quality-assured products
Bangladesh	Low	7	–	34	1^a^	13	–
Liberia	Medium	2	–	2	1^a^	–	–
Malawi	High	–	–	5	3^a^^,b,c^	–	–
Nepal	Low	5	1^d^	4	–	3	–
Nigeria	High	3	–	12	2^a^^,c^	–	–
Rwanda	Medium	–	–	–	–	–	–
Sierra Leone	High	2	–	2	1^a^	–	–
South Africa	Low	–	–	1	1^c^	2	2^e,f^

^a^Misoclear®/Mistol®/Jekprostol®/, Acme, WHO PQ-listed

^b^Misoprost®, Cipla Pharmaceuticals, WHO PQ-listed

^c^Cytotec®/, Pfizer Pharmaceuticals (This is the originator misoprostol product and is not SRA-approved for medical termination of pregnancy only the prevention and treatment of gastric ulcers)

^d^Medabon®, Sun Pharmaceuticals, SRA-approved

^e^Mifegyne®, Exelgyn Pharmaceuticals, SRA-approved

^f^Mediprist®, LinePharma, SRA-approved

### Policy and financing

Standard treatment guidelines (STG) indicate rational and judicious use of medicines for specific health indications and are recommended to be updated concurrently with national essential medicines list (NEML) which prioritize medicines to be procured for the public sector healthcare system [12]. National abortion care guidelines define who, when, where, and how safe abortion services are delivered in the country. The inclusion of combination regimen in NEML, STGs and abortion care guidelines varied across the countries assessed (Table 2). Mifepristone and misoprostol regimen for induced abortion was identified on the NEML/STG for South Africa (2019) as well as in specific abortion care service and delivery guidelines for Bangladesh, Nepal, Nigeria, and Rwanda [13–17]. Neither Liberia, nor Malawi included mifepristone and misoprostol combination regimen on its NEML/STGs but included misoprostol for postpartum hemorrhage (PPH) and postabortion care (PAC) management. Nigeria’s 2nd edition STG (2016) and Sierra Leone’s *National Protocols and Guidelines for Emergency Obstetric and Newborn Care* (2018) included misoprostol for PPH and PAC. At the time of the assessment, mifepristone’s inclusion was being considered for the Nigeria NEML and the combination regimen was added in 2020 [18]. In all countries, the use of misoprostol for PPH and PAC was included in the NEML/STG and/or service delivery guidelines.

**Table 2:** Extent to which MA commodities or protocols for their use were included in policy documents

Country	Abortion law restrictions category	MA medicines on NEML (Year)	Type of guideline that specifies MA protocols
Bangladesh	Low	None (2016)	Abortion care service and delivery guidelines^*^
Liberia^±^	Medium	Misoprostol (2017)	None
Malawi^±^	High	Misoprostol (2015)	None
Nepal	Low	Mifepristone & Misoprostol (2016)	Abortion care service and delivery guidelines
Nigeria	High	Misoprostol (2018)^^^	Abortion care service and delivery guidelines
Rwanda	Medium	Mifepristone & Misoprostol (2015)	Abortion care service and delivery guidelines
Sierra Leone	High	Misoprostol (2016)	None
South Africa^±^	Low	Mifepristone & Misoprostol (2012)	Standard Treatment Guidelines

^*^Menstrual regulation in Bangladesh

^±^National Essential Medicines List and Standard Treatment Guidelines are combined in a single document

^At the time of the assessment, mifepristone was not included on the NEML but under consideration. The combination regimen was added in 2020

### Procurement and distribution

Procurement is the process by which a government acquires needed products and services by purchasing from commercial businesses, in this case, manufacturers and/or wholesale distributors of MA medicines. The government stores these products in their central medical stores departments at the national and/or provincial level and then distributes these products to public sector facilities. We identified whether public sector tenders and procurement of MA medicines had occurred at least once in the past 3 years preceding the assessment. Products that were listed on the NEML were also procured for the public sector at least once (Table 3). In all countries misoprostol had been procured for PPH and PAC at least once in the past 3 years for the public sector; in Nepal, Rwanda and South Africa, public sector tenders for either a combi-pack product or mifepristone were identified.

**Table 3:** Public sector tenders and procurement for MA medicines

Country	Misoprostol on NEML	Misoprostol procured	Combination regimen on NEML	Combination regimen Procured
Bangladesh	–^±^	Yes	–	–
Liberia	Yes	Yes	–	–
Malawi	Yes	Yes	–	–
Nepal	Yes	Yes	Yes	Yes
Nigeria	Yes	Yes	–	–
Rwanda	Yes	Yes	Yes	Yes
Sierra Leone	Yes	Yes	–	–
South Africa	Yes	Yes	Yes	Yes*

^±^The current Bangladesh NEML 2016 assessed, excluded misoprostol; the previous edition (2008) included misoprostol

^*^Mifepristone and Misoprostol procured separately, not as a combi-pack

MA was purely a private sector commodity in Liberia, Sierra Leone, and Nigeria, where social marketing organizations (SMOs) had registered and imported combi-pack but were limited in their distribution owing to a restrictive policy environment and/or lack of safe abortion service delivery guidelines (Table 2).

Rwanda and Nigeria illustrate ways in which the combination regimen was added to the NEML despite more restrictive abortion laws. In Rwanda, the government took the pragmatic step to add mifepristone and misoprostol to the 2015 NEML to align with the revised penal code of 2012 which permitted abortion resulting from rape, incest, forced marriage, or on medical grounds [19]. Rwanda wanted to have all WHO recommended abortion methods [2] available in such cases and the NEML application was accepted. In Nigeria, at the time of the assessment, a multi-stakeholder group of NGOs and researchers had engaged in consensus building with the NEML committee, presenting information on the safety, efficacy, stability, and pharmacodynamics of the MA medicines. During this time, the NRA had approved three MA products for registration, the government had approved the *National Guidelines on Safe Termination of Pregnancy for Legal Indications*, and the WHO had added the combination regimen to its Model List of Essential Medicines (2019). Bolstered by these events, it was added to the NEML in 2020 [18].

We found that adding a MA medicine to the NEML does not guarantee that once procured, distribution will happen readily. In both Rwanda and South Africa, key informants shared that central medical stores staff delayed distribution or locked up MA medicines citing concerns that MA medicines will be “misused.” In South Africa and Nepal, procurement is decentralized to the provincial level and determined by each provinces’ public health prioritization and budgets for commodities.

In all countries, funding for medicines was also an issue. In several countries, budget constraints required governments to re-prioritize procurement lists to a quarter of those deemed essential, and MA commodities were often eliminated, despite being on the NEML. As such, stock outs of MA medicines persist. In such cases, donors like UNFPA, or SMOs, were asked to procure misoprostol and/or combi-packs for public sector distribution.

*“Adding another product to the EML is like creating a wish list for shopping. As a government we don’t even have the funds to procure everything that is already on the list, why add another item? It would cost $22 million to purchase everything on the EML to meet the entire country’s need.”* – *Key informant, Liberia*

### Provider knowledge

Provider knowledge was assessed using proxies such as availability of ministry-approved training manuals and curricula and documented training efforts of healthcare workers (Table 4). In Bangladesh, Nepal, Rwanda and South Africa, provincial and/or national governments had supported a limited number of in-service trainings of public sector providers on abortion care, including MA. In Liberia, Malawi and Sierra Leone—countries with highly restrictive abortion laws and no abortion service delivery guidelines or training curricula—no government-supported training on medical abortion for public sector providers had occurred. Instead, training on MA was either limited in scope to selected private sector providers and pharmacists, or prohibited. This created a bottleneck to service provision and also meant that the product risked expiring before use. Nigeria proved to be an exception; following the development of ministry-approved guidelines on abortion provision within the legal framework in 2019, the Society of Obstetricians and Gynecologists of Nigeria trained 18 master trainers on comprehensive abortion care (CAC), across all six country zones, with future cascade trainings planned. CAC includes the provision of information, abortion management (including induced abortion), and care related to pregnancy loss/spontaneous abortion and PAC [2].

**Table 4:** Provider training efforts on MA medicines

Country	Nationally-approved in-service training curricula including MA	Pre-service curricula including MA protocols	Public sector in-service training on MA	Private sector in-service training on MA
Bangladesh	Yes	–	Yes	–
Liberia	–	–	–	–
Malawi	–	–	–	–
Nepal	Yes	Yes	Yes	Yes
Nigeria	Yes	Yes	Yes	Yes
Rwanda	Yes	–	Yes	Yes
Sierra Leone	–	–	–	Yes
South Africa	–^±^	–	Yes	Yes

^±^New National Clinical Guidelines for Implementation of the Choices on Pregnancy Termination Act provided the basis for developing a national training curriculum on safe abortion after the assessment

Where trainings had occurred in the public sector (Bangladesh, Nepal, Nigeria, Rwanda, and South Africa), key informants reported poor coordination between government training efforts and central medical stores’ distribution supply chain. This sometimes resulted in a lack of MA medicines at the facilities with trained staff. In the case of Rwanda, this situation was exacerbated by no registered MA products in the private sector. Once initial donated program drug stocks were depleted in the facilities, trained providers lacked access to MA drugs because a prescription was unable to be filled at an outside pharmacy.

We found a paucity of nationally approved pre-service curricula including MA in schools of medicine, midwifery and nursing (Table 4). Training on mifepristone and misoprostol for medical students was also limited. For instance, in 2010 in Nigeria, Ipas supported pre-service education on CAC in ten medical colleges, which included medical methods, but pre-dated the registration of combi-pack or mifepristone in Nigeria. In Nepal, pre-service training on abortion is mixed across healthcare cadres and schools. At the time of the assessment, WHO Nepal was reviewing existing curricula on abortion care to inform the development of government-approved standardized pre-service curricula on CAC, including MA for medical, midwifery and nursing schools. Few countries assessed were maximizing WHO healthcare worker guidance related to abortion service provision which limited abortion services to only doctors and specialists at the highest-level facility in their country.

Interviews with key informants, some of whom were providers themselves, suggested that some healthcare providers lacked knowledge of their country’s abortion law. Lack of awareness about the medical indications that would permit therapeutic abortion, fear of litigation, and deeply entrenched abortion-related stigma influenced providers’ willingness to offer services and commercial distributors’ interest to stock or promote MA products. In some of the countries assessed, providers in positions of influence at teaching and referral hospitals and professional associations held negative views of abortion.

*“Here we have many issues of heads of hospitals not always being aware of the conditions whereby women and girls can get a legal abortion. I was at a site visit at a large district hospital once and the midwife in charge of the maternity ward was unaware that a court order was no longer required in cases of rape and incest, and that any girl under 18 years old can receive abortion on demand with presentation of her ID. This was more than 6 months after the revised penal code had been in the Gazette.”—Key Informant, Rwanda*

### End-user knowledge

A review of the literature showed that even in settings where abortion is broadly legal, most women do not know that it is an option. This was the case in Bangladesh, Nepal and South Africa, where less than half of women surveyed knew abortion was legally available in their country [20–22]. For those that did, they sought abortion through a variety of means, including at health facilities, traditional healers, pharmacies, and clinics [5, 23–25]. In South Africa, women were also accessing pills online [25].

In countries where abortion was more restricted (Liberia, Malawi, Nigeria, Sierra Leone), there was little data on the incidence of unsafe abortion and women’s knowledge of the abortion law or services. In these countries, key informants widely believed that abortion stigma was common and driving the practice towards less safe methods, contributing to preventable death and disability, particularly among adolescents and rural populations. Private sector pharmacists interviewed often expressed that they were the first point of contact for those seeking assistance with an unwanted pregnancy.

*“Education levels are low, the population is young and the government doesn’t realize we are doing them a favor by making family planning, emergency contraception and MA products available in pharmacies—because that is where the youth will go first, not a facility, not their parents.” -Key Informant, Liberia*

Community awareness activities on MA have been limited in scope across the countries assessed. Small-scale efforts to utilize mobile health and/or sensitize communities on abortion services via helplines and community health workers were being utilized in Bangladesh, Nepal, Nigeria, Rwanda, and South Africa. It was also loosely understood that informal networks, hotlines, pharmacies, and word of mouth play a role in women’s knowledge and access to abortion, including MA in the private sector. We found that SMO’s typically included helpline numbers on MA product inserts as a strategy to educate women on correct use and management.

## Discussion

This paper describes the results of an eight-country assessment aimed to understand the factors that influence availability of MA medicines. Our assessment reinforced findings from other market shaping reports that the legal status of abortion does not determine the ability to register MA medicines [26, 27]. Registration of MA medicines—misoprostol or mifepristone and misoprostol, was established in all countries assessed, except Rwanda. However, of MA medicines that are approved by an SRA or are WHO PQ-listed, only Nepal and South Africa had quality-assured combi-pack and mifepristone products registered, respectively. The distinctiveness of NRAs from other government agencies has meant that the medicine regulatory process is more immune to abortion politics and registrations of misoprostol and the combination regimen have expanded in countries with different legal frameworks. NRA’s reliance upon evidence-based practice, often using the Common Technical Dossier (CTD) format that focuses on review of the scientific data on the medicine’s quality, safety and efficacy, as well as laws governing pharmaceutical supply, storage and distribution, has been documented in other low- and middle-income countries [28–30]. Through an independent, scientifically-driven process NRAs in highly abortion restrictive countries like Liberia, Nigeria and Sierra Leone, approved multiple MA products for license, import and dispensing on prescription through private sector points of consumption and sale, including private pharmacies, clinics, and hospitals. However, despite existing “fast-tracked” application processes, NRAs can be slow to approve medications, lacking regulatory capacity and transparency in decision-making [31, 32].

As such the assessment identified the opportunity to support in-country registration of additional mifepristone and misoprostol products that are approved by an SRA or WHO PQ-listed, irrespective of abortion laws, utilizing the WHO Collaborative Registration Procedure process or other regional regulatory reliance mechanisms to increase the availability of quality-assured products.

We found that in countries with highly restrictive abortion laws, the legal framework inhibited the development of policies and practices that would increase access to MA medicines and safe abortion services; such as inclusion in STG and national abortion care service delivery guidelines, NEMLs and public sector procurements. The exception was Nigeria, where despite a restrictive abortion law, the development of national abortion care guidelines, enabled both the addition of mifepristone on the EML and public sector training of master trainers on CAC in each region of the country. The existence of clinical practice guidelines has been shown to improve the provision and quality of care [33, 34]. Health worker knowledge about abortion legislation and services has been shown to be one of several key components to successful implementation of service provision [35, 36]. We found that a lack of provider knowledge of the legal framework for abortion impacts service delivery and demand for MA. Additionally, providers’ beliefs, biases and fear of litigation, influences their willingness to offer services, as has been shown in other countries [3, 37–39].

The assessment highlighted the need to support the development of government-approved and validated CAC guidelines to maximize the operationalization of the law in each country context. UN agencies; international NGOs, professional organizations and other organizations with technical expertise can support governments to draft, validate and disseminate updated evidence-based practices to define who, where, when and how abortion care can be offered. Even in countries where guidelines exist (Bangladesh, Nepal, Nigeria, and Rwanda), they are not readily available at the district level and at the facilities.

Our assessment showed that inclusion on the NEML is an important driver of public sector procurement of MA medicines. Misoprostol is more commonly included on the NEMLs in the countries we assessed and had been procured in every country at least once. This is consistent with the WHO Global EML Database of 137 countries that shows misoprostol has been included on NEMLs with greater frequency (n = 86) than in combination with mifepristone (n = 16) in the past decade [40]. This is likely attributable to the fact that misoprostol, which may be used alone for MA, has been listed on the WHO Model List of Essential Medicines for management of PPH, PAC and labor induction since 2011 [2, 41]. Considering misoprostol’s multiple obstetric and gynecologic uses and its broader availability than the combi-pack, the misoprostol-only regimen will likely continue to be an important method to address induced abortions [42]. Where MA medicines were included on the NEML, the government had a rationale for public sector procurement and tenders for both mifepristone and misoprostol had been issued in the three countries that included them on their NEMLs. However, countries also need reliable financing for procurement of essential medicines to guarantee public sector supply, something that our assessment found lacking in a number of countries. WHO’s 2020 guide, *Selection of Medicines at Country Level*, outlines steps countries should undertake to develop and update their NEMLs and increase their capacity to reimburse payments for medicines [12].

The assessment identified that many countries have the opportunity to strengthen public sector procurement mechanisms for the combi-pack, including adding the combination regimen to NEMLs, defining funding for MA commodities based upon government and donors’ funding streams, improving upon facility documentation of PAC and abortion cases to improve forecasting and quantification of need, and orienting central medical stores staff to policies regarding MA medicines to ensure distribution of stocks in a timely manner. Countries classified as low-income by the World Bank (Liberia, Malawi, Nepal, Nigeria, Rwanda and Sierra Leone, in our sample) are also eligible to sign agreements with UNFPA’s Supplies Partnership 2021–2030 that offer access to competitive negotiated prices on commodities, low overhead rates, bridge funding for commodities, and capacity-building in procurement processes [43].

Providers serve as important gate keepers to MA commodities and services. Our assessment showed that where national guidelines existed, abortion service provision was more likely to be included in the public sector healthcare system at some level (e.g. a reference or teaching hospital). Training on MA is a significant bottleneck to service provision and access to MA medicines. Few countries had pre-service curricula that included MA and integrating standardized pre-service curricula has been shown to enhance training efforts [44, 45]. Even in countries such as Bangladesh and Nepal that have implemented CAC training programs, staff turnover, secondments, and retirements can result in service delivery gaps [46].

The assessment identified the opportunity in all countries to maximize WHO healthcare worker guidance related to abortion provision and establish public–private partnerships to provide large-scale training of mid-level public providers and pharmacists on MA. In particular, professional medical societies need support to update pre-service training curricula to include misoprostol and the combination regimen for induced abortion in medical, nursing and midwifery colleges and address values clarification and conscientious objection among their constituents. Models such as Nepal’s, which designate free, branded, government-run Safe Abortion Service centers should be replicated. Moreover, there is the opportunity through mobile and digital platforms to expand mentorship programs and networks of safe abortion providers.

Studies have shown that women’s knowledge of their abortion rights in the countries assessed is low [18, 20, 47–49]. Stigma, poverty, knowledge of and distance to services and pharmacies all have been shown to impact women’s access to abortion [3, 19, 49, 50]. We found that in most countries, community awareness campaigns about abortion were very limited, despite the establishment of supply.

The assessments identified the need for more direct-to-consumer community awareness campaigns, optimizing digital platforms, mobile health and informal networks. Specifically, campaigns to inform end-users about their rights within the legal framework, the availability of safe abortion services in the private and public sector or accurate mobile-based technologies for information and counseling on MA to influence consumer behavior must be prioritized. Models that utilize mobile health to reach women, providers and pharmacists alike should be expanded.

### Strengths and limitations

A strength of the assessments is that the holistic approach is useful to governments and program partners who may only be active in one component of availability. Importantly, we assessed both the commodity supply-side components with provider and end-user knowledge. These latter two components ensure acceptance, demand for and adoption of a health commodity at the facility and community-levels. The national landscape assessments present some limitations. We limited our inquiry to those products formally registered by the NRA of each country. Assessing the availability of unregistered products, pricing and prescription-status was outside the scope of the assessments but is documented elsewhere [7]. Provider and end-user knowledge were assessed by proxies relying on the available published literature and key informant interviews, instead of formal knowledge, attitudes and practices surveys. While some tangible data could be collected on the existence of service delivery guidelines and training efforts in each country that influenced provider knowledge, we acknowledge that assumptions about end-user knowledge of their legal right to abortion and ability to access services is simplistic. We acknowledge that end-user knowledge relies upon other important potential barriers such as knowledge of where to access services and medicines, transportation, cost of services and medicines, and the quality of information and counselling received, which is documented elsewhere [38, 51, 52]. We used a sample of key informants based upon interviewees’ availability during the rapid in-country assessments. As such, in a few countries some interviews with key ministry staff or non-government organization partners could not be secured and we had to rely on published literature or reports. However, these assessments were not meant to generate generalizable knowledge, but rather to serve as a resource for countries to develop actionable strategies. Despite these limitations, several cross-cutting opportunities that are impacting the availability of MA in each country context were identified.

## Conclusion

Understanding the factors that influence the availability of MA medicines is important to help policymakers and program planners in countries improve availability of medicines. Our national landscape assessments utilized a framework that includes both supply and demand sides of commodity availability, taking into account the interplay of factors from product introduction to use. The national landscape assessments can serve as a resource for countries to develop actionable strategies to ensure availability of quality-assured MA medicines.

## Data Availability

The data that support the findings of this study are available from the corresponding author, URL, upon reasonable request.

## Abbreviations

CAC: Comprehensive abortion care
CTD: Common Technical Dossier
MA: Medical abortion
NEML: National Essential Medicines List
NRA: National regulatory authority
PAC: Post-abortion care
PPH: Postpartum hemorrhage
SMO: Social marketing organizations
SRA: Stringent regulatory authority
STG: Standard Treatment Guidelines
WHO PQ: WHO Prequalification
