# Correction: Availability of medical abortion medicines in eight countries: a descriptive analysis of key findings and opportunities

**DOI:** 10.1186/s12978-023-01691-z

**Published:** 2023-10-26

**Authors:** Amy Grossman, Ndola Prata, Natalie Williams, Bela Ganatra, Antonella Lavelanet, Laurence Läser, Chilanga Asmani, Hayfa Elamin, Leopold Ouedraogo, Md. Mahmudur Rahman, Musu Julie Conneh-Duworko, Bentoe Zoogley Tehoungue, Harriet Chanza, Henry Phiri, Bharat Bhattarai, Narayan Prasad Dhakal, Olumuyiwa Adesanya Ojo, Kayode Afolabi, Theopista John Kabuteni, Binyam Getachew Hailu, Francis Moses, Sithembile Dlamini-Nqeketo, Thembi Zulu, Ulrika Rehnström Loi

**Affiliations:** 1 Venture Strategies for Health & Development/OASIS, Berkeley, CA USA; 2 Bixby Center for Population, Health & Sustainability, School of Public Health, University of California, Berkeley, CA USA; 3 UNDP-UNFPA-UNICEF-WHO-World Bank Special Programme of Research, Development and Research Training in Human Reproduction (HRP), Department of Sexual and Reproductive Health and Research, World Health Organization, 20 Avenue Appia, 1211 Geneva, Switzerland; 4 World Health Organization, Regional Office for Africa, Brazzaville, Republic of Congo; 5 Directorate General of Family Planning, Karwarbazar, Dhaka Bangladesh; 6 World Health Organization, Liberia Country Office, Monrovia, Republic of Liberia; 7 Family Health Program, Ministry of Health, Monrovia, Republic of Liberia; 8 World Health Organization, Malawi Country Office, Lilongwe, Republic of Malawi; 9 Ministry of Health, Lilongwe, Republic of Malawi; 10 Department of Drug Administration, Ministry of Health and Population, Kathmandu, Nepal; 11 Ministry of Health and Population, Kathmandu, Nepal; 12 World Health Organization, Nigeria Country Offce, Abuja, Federal Republic of Nigeria; 13 Reproductive Health, Federal Ministry of Health, Abuja, Federal Republic of Nigeria; 14 World Health Organization, Rwanda Country Office, Kigali, Republic of Rwanda; 15 World Health Organization, Sierra Leone Country Office, Freetown, Sierra Leone; 16 Reproductive Health/Family Planning Programme Manager, Ministry of Health, Freetown, Sierra Leone; 17 World Health Organization, South Africa Country Office, Pretoria, Republic of South Africa; 18 National Department of Health, Pretoria, Republic of South Africa

**Correction: Reproductive Health (2023) 20:58** 10.1186/s12978-023-01574-3

After publication of this article [1], the authors reported that the Acknowledgements section should have read ‘The authors acknowledge the role of Sarah Jones at VSHD/OASIS for conducting the country assessments and compiling the report findings with the help of in-country consultants, Eugène Kanyamanza, Evangeline Dushimeyesu, Gabriele von Wahlert, Philip Dorrell, and Sameena Chowdhury and as well as the collaboration of key informants who shared their valuable knowledge with the authors.'

The original article [1] has been corrected.
